# Versatile platform of nonlocal metasurfaces for both spectral and spatial control of light waves

**DOI:** 10.1038/s41377-022-00986-3

**Published:** 2022-10-11

**Authors:** Run Chen, Shuming Wang

**Affiliations:** National Laboratory of Solid-State Microstructures, School of Physics, Nanjing University, Nanjing, 210093 China

**Keywords:** Metamaterials, Displays

## Abstract

Multifunctional nonlocal metasurfaces based on quasi-bound states in the continuum are experimentally demonstrated, which shapes the wavefronts at the resonant wavelengths while have no effect on other wavelengths. By means of cascade and orthogonal perturbations, the nonlocal metasurface devices can be extended to a versatile platform with multifunction.

To date, metasurfaces have become a platform for controlling the phase, amplitude, polarization and chromatic aberration of light waves^1–5^. Generally, according to the interaction types between adjacent elements, metasurfaces can be divided into two types. One is the local metasurfaces with no interaction between adjacent metaunits, which have advantages in wavefront shaping (spatial control) but lack the ability of frequency selectivity (spectral control) due to the wideband response. The other one is nonlocal metasurfaces with interactions between adjacent metaunits, supporting a collective mode^6,7^. Traditional nonlocal metasurfaces exhibit effective frequency selectivity, but lack of spatial control of light^8^.

In order to effectively manipulate light wave in both spectral and spatial dimensions, a nonlocal metasurface designed with phase-gradient guided mode resonant grating is proposed to control the wavefront at the resonant wavelength^9^. However, the flexibility of wavefront shaping of the device is limited to high deflection angles in a single fixed direction relative to the grating. To further improve the ability of nonlocal metasurface in phase control, Overvig et al. successfully introduced the concept of spatial geometric phase into the non-local metasurfaces supporting quasi-bound states in the continuum (q-BICs), and theoretically demonstrated the multifunctional nonlocal metasurfaces for independent wavefront shaping at resonant wavelengths^10,11^. It is proved that the flexible spectral and spatial control of light wave can be simultaneously realized by the nonlocal metasurfaces.

In recent work published in *Light: Science & Applications*, Malek et al. experimentally demonstrated a multifunctional platform of nonlocal metasurfaces in the near-infrared region for independent multispectral wavefront shaping with high Q-factors^12^. The systems are based on photonic crystal slabs fabricated on an amorphous silicon film etched with pairs of rectangular holes on silica substrate supporting q-BICs encoded with a spatially varying geometric phase. These nonlocal meta-devices, including a nonlocal radial metalens, a dual-function cylindrical metalens, and metalens doublets with up to four distinct functionalities, shape optical wavefronts at the resonant wavelengths, leaving the other wavelengths unchanged, with circularly polarized light incidence.

In this article, the authors fabricated a nonlocal radial metalens with NA = 0.2 and a diameter of 800um, which focuses optical wavefront at the resonant wavelength of *λ* = 1590 nm with almost undetectable focusing efficiency at other wavelengths. Due to the carefully designed meta-unit library, the resonant wavelength across the entire metalens is nearly constant, which is confirmed by the Strehl ratios of 0.89 and 0.85 in the x and y directions, respectively. To achieve the multifunctional nonlocal meta-optics systems, two approaches are adopted, which are cascading nonlocal metasurfaces with distinct resonant wavelengths and adding to a single-layer metasurface a set of orthogonal perturbations independently controlling the corresponding q-BICs. For the former method, a nonlocal metalens doublet is fabricated, consisting of a converging cylindrical metalens, generating a focal line, and a diverging radial metalens sharing the same focal plane located between them at resonant wavelength of *λ* = 1450 nm and *λ* = 1590 nm, respectively. For the latter one, a single-layer multifunctional metasurface with orthogonal perturbations controlling targeted q-BICs is fabricated. The designed meta-device works as two orthogonal cylindrical lenses generating two orthogonal focal line at the same focal plane at the resonant wavelength of *λ* = 1385 nm and *λ* = 1460 nm, respectively.

For more highly multifunctional metasurfaces, the two approaches mentioned above can be combined together. As shown in Fig. 1, a three-function nonlocal metasurface doublet (Fig. 1a) and a four-function one (Fig. 1b) are fabricated as a proof of principle. The three-function device is consisted of a dual-function cylindrical lens at two resonant wavelengths of *λ* = 1428 nm and *λ* = 1486 nm, and a single-function diverging lens at resonant wavelengths of *λ* = 1620 nm. These three lenses share the same focal plane between two cascading nonlocal metasurfaces with Q-factors from ~60 to ~90. A four-function doublet is also fabricated, which is cascading with a dual-function cylindrical lens and a dual-function diverging cylindrical lens, sharing the same focal plane at the targeted wavelengths with Q-factors from ~100 to ~300. Furthermore, the application of this multi-functional non-local metasurface in augmented reality glasses (AR-glasses) are finally proposed. By means of orthogonal perturbations and dual-layer cascade, the wavefront control of red, green and blue (RGB) wave is realized. This device can reduce the influence of ambient light as much as possible, and is an effective design scheme for the optical components of AR-glasses.

**Fig. 1:**
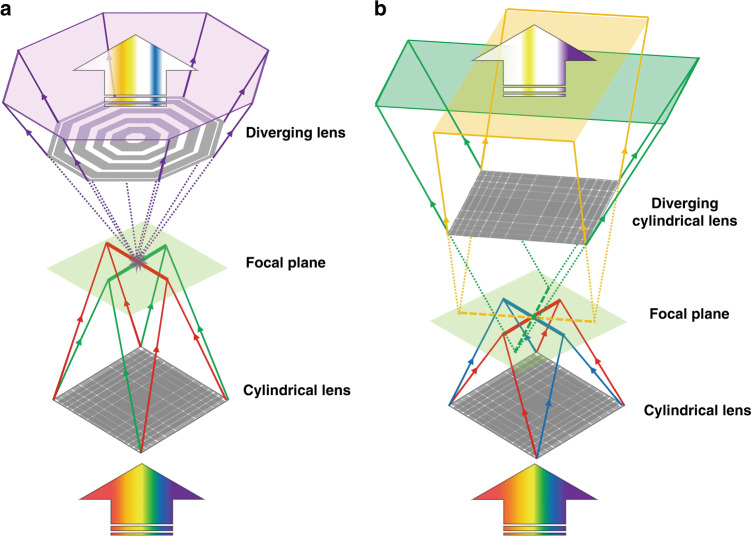
Schematic diagram of multifunctional platform of nonlocal metasurfaces. **a** Schematic of a three-function doublet composed of a single-function diverging metalens and a dual-function converging cylindrical metalens. **b** Schematic illustrating a four-function doublet composed of a dual-function diverging cylindrical metalenses and a dual-function converging cylindrical metalenses

Through this work, the authors have successfully achieved a versatile platform that can effectively manipulate the wavefront of light at the targeted wavelengths with steerable Q-factors. Compared with the previous work, the system has better performance in spectral and spatial control. The functions of the optics system can be easily expanded by means of multi-layer cascade, orthogonal perturbations and the combination of the two methods.

In future work, on the one hand, it is important to search for new physical mechanisms to deal with the inefficiency of this scalable system, such as the introduction of chirality. On the other hand, for further extension of the functionalities, the tunability of the platform is also a worthwhile goal to pursuing. This nonlocal metasurface platform can be used in many fields with its efficient frequency selectivity, wave-front control ability and strong light-matter interaction, such as nonlinear optics, quantum optics and so on.
